# Signed in bone: Toolmark identification of a machete and multidisciplinary 3D reconstruction in a homicide investigation

**DOI:** 10.1016/j.fsisyn.2026.100739

**Published:** 2026-09-18

**Authors:** Melissa Kirbach, Anna Holzer, Larissa Frey, Anna Tegou, Sven Helmrich, Markus Alexander Rothschild, Matthias Weber

**Affiliations:** a Institute of Legal Medicine, Medical Faculty, University of Cologne, Melatengürtel 60/62, Cologne, 50823, Germany; b Institute of Legal Medicine, University Hospital Essen, University Duisburg-Essen, Essen, Germany; c Landeskriminalamt Nordrhein-Westfalen (LKA NRW) - Institute of Forensic Science, Düsseldorf, Germany

**Keywords:** Interdisciplinary approach, Tool marks, 3D reconstruction, Maceration, Sharp force trauma, Homicide investigation, Cross-correlation

## Abstract

This study presents a homicide case involving sharp force trauma. A chop mark in the parietotemporal skull provided very strong support for the proposition that it was produced by the seized machete, based on conventional comparison microscopy and optical comparison of 3D surface scan data. This result was further supported by quantitative comparison using normalized cross-correlation (XCorr = 0.73) of the striated mark in the bone and the produced test marks of the machete. By integrating findings from toolmark examination, forensic pathology, postmortem CT (pmCT) imaging, bloodstain pattern analysis, and crime scene documentation, a three-dimensional reconstruction of the moment of impact was created. This case demonstrates the value of toolmark analysis of sharp force trauma in homicide investigations. It also highlights the potential of integrating multiple forensic disciplines to generate comprehensive three-dimensional reconstructions of violent crimes for use in criminal investigations and the presentation of forensic evidence in court.

## Highlights

• An interdisciplinary approach enhanced the evidential value and supports the presentation of forensic evidence in court.
• Toolmarks provided very strong support for the proposition that they were produced by a seized machete.
• Optimized Cross-correlation analysis of the striated mark (bone) and the test mark (machete) supported the findings.
• pmCT, forensic authopsy and bloodstain pattern analysis enabled 3D crime scene reconstruction.

## Graphical abstract

**Figure:**
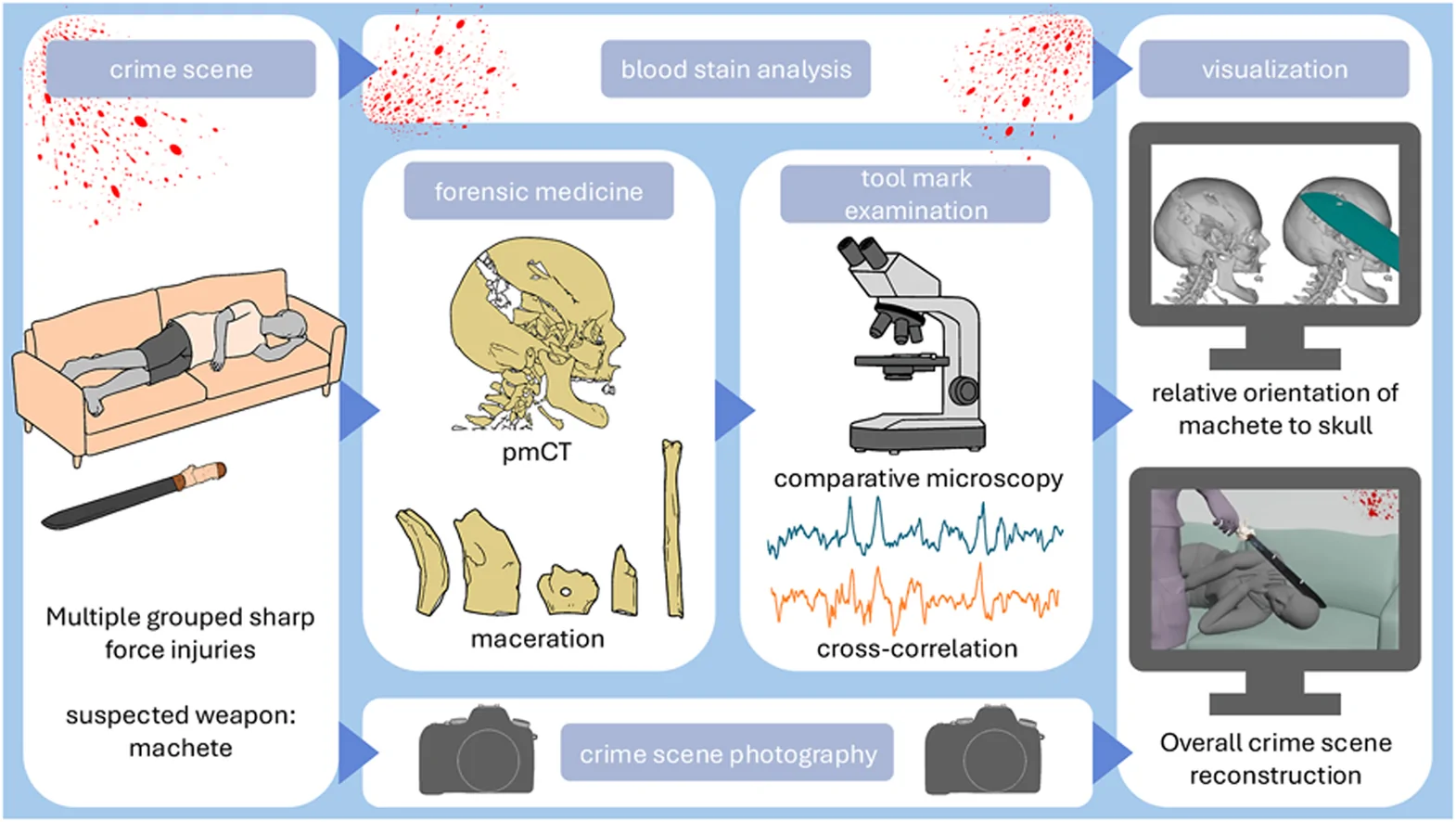

## 1 Case background

An individual was found lying on a sofa without signs of life and exhibiting multiple sharp force injuries to the head and upper extremities. The body was positioned resting on the left side. Despite the arrival of emergency medical services, death was pronounced at the scene. A considerable amount of blood was present particularly on and around the upper body, in the region of the head and upper extremities. In contrast, only minimal blood staining was observed on the abdomen, trousers, and feet.

According to the attending emergency medical personnel, the original position of the body was altered during resuscitative and first-aid measures.

## 2 Forensic medical examination and evidence collection

**Fig. 1:**
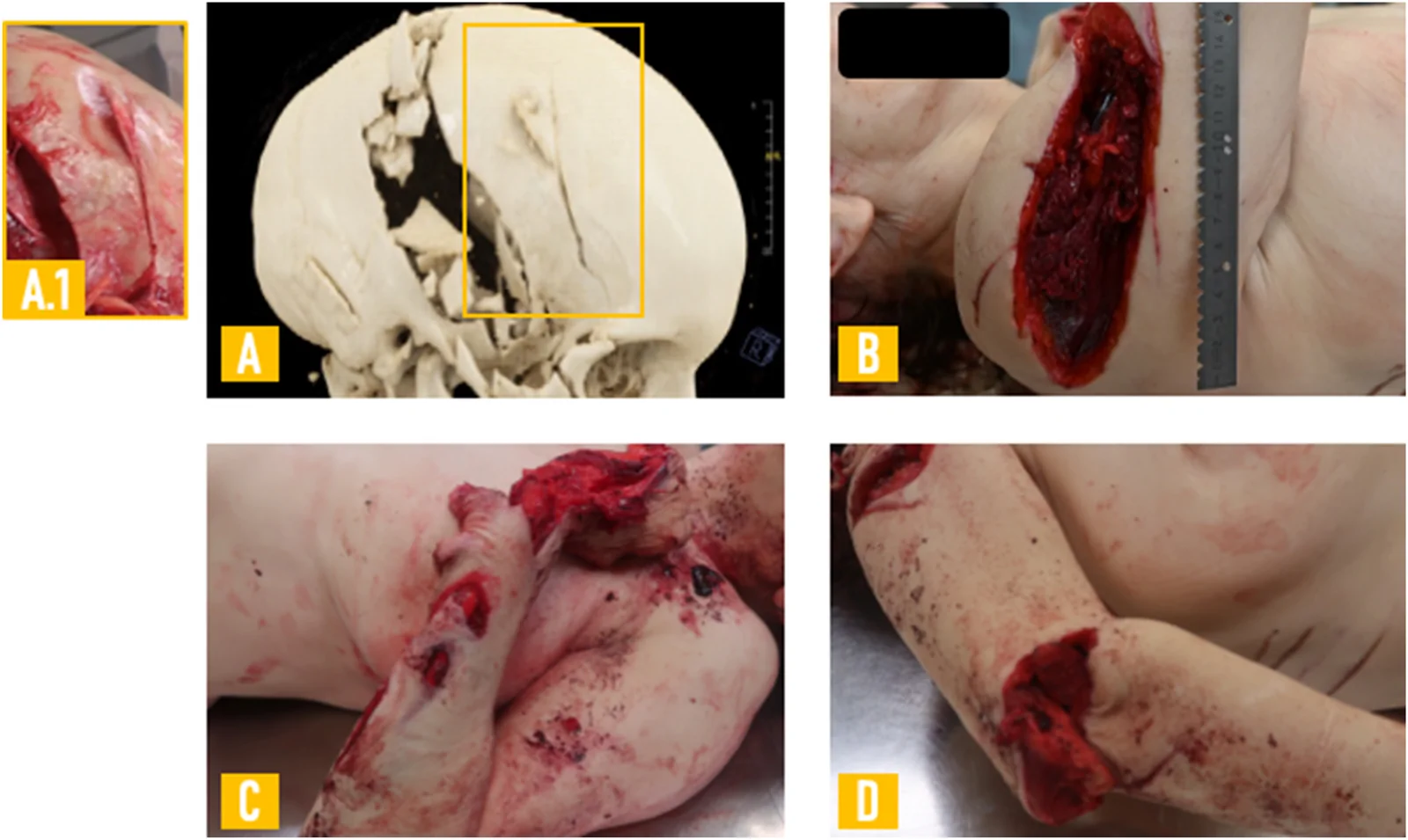
Injuries caused by sharp force trauma. A 3D visualization of the pmCT data of the complex open right temporoparietal skull fracture with A.1 Detailed photograph of the tool mark suitable for examination. B Right upper arm with fracture of the humerus C Left forearm with fractures of the ulna, carpal bones, and metacarpal bones D Right elbow joint with fractures of the humerus and ulna.

The individual was of middle age, weighed approximately 55 kg, and measured 168 cm in height. Autopsy and postmortem CT (pmCT) findings revealed multiple grouped sharp-force injuries to the right side of the head (Fig. 1, A), resulting in skull fractures and extensive destruction of brain tissue. One of the tool marks on the cranial bone was considered suitable for further examination (Fig. 1, A1). Additional sharp-force injuries were identified on the upper extremities, accompanied by fractures of multiple long bones (among other, on the right elbow joint, the right humerus, and the left ulna, Fig. 1, B–D). The cause of death was determined to be severe open craniocerebral trauma in combination with massive blood loss.

**Fig. 2:**
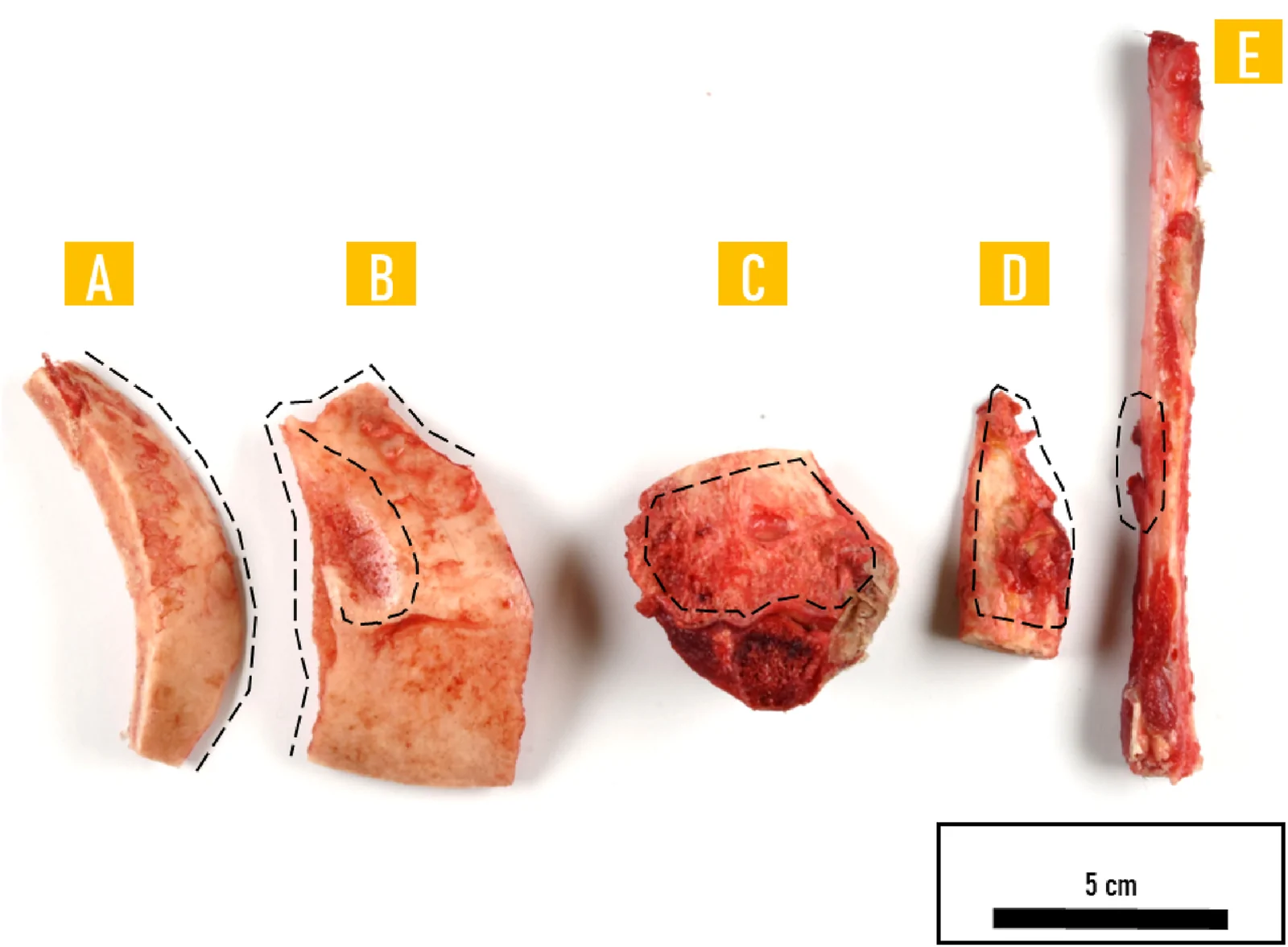
Bone fragments; relevant potential marks and material are identified and labeled according to their assessed origin (autopsy-related vs. crime-related). The dashed lines indicate areas containing potential tool marks; A, B skull bone fragments, C right elbow joint fragment, D right humerus fragment, and E left ulna fragment.

For subsequent toolmark analysis, a section of the skull bone as well as samples of the right elbow joint, right humerus, and left ulna were excised using an oscillating saw and submitted, in a cooled state, to the forensic science institute in charge of the analysis. To enable subsequent examiners to distinguish between marks produced during the autopsy and toolmarks relevant to the crime, all bone fragments were jointly reviewed by the forensic pathologist and the toolmark examiner. The marks were discussed with regard to their origin and subsequently identified and labeled accordingly by the forensic pathologist (Fig. 2).

## 3 Initial assessment

The bone fragments of the skull, right elbow joint, right humerus, and left ulna were initially examined under a light microscope for potential microtraces; none were detected. The bone fragments were examined for the presence of evaluable tool marks, with particular emphasis on chop marks. During this examination, one of the preserved cranial bone fragments exhibited a chop mark that was classified as potentially suitable for further forensic evaluation. This assessment was based on the presence of clearly discernible and well-preserved striations within the mark. In contrast, the marks on the right elbow joint, right humerus, and left ulna were classified as having limited potential for further forensic evaluation, as they exhibited striations of limited quantity and detail, thereby restricting maceration.

The specimens subsequently underwent maceration in a water bath without the addition of detergents, enzymes, or other chemical agents. In the forensic context, maceration denotes the controlled removal of soft tissue by soaking [1] from skeletal elements in order to expose osseous structures for examination. For this purpose, each bone specimen was placed individually in a gauze bag and macerated at a maximum temperature of 65 °C for up to 16 days (detailed process in the Supplementary Material Fig. A1).

The bone fragments underwent alternating cycles of warm-water maceration at 60 °C and cold water-watering rinsing to remove loose tissue while minimizing the applied pressure to prevent alteration of traces. The bone fragment of the right elbow joint needed most time for the complete maceration since the attached tendons as well as the present articulate cartilage are more resistant to degradation and therefore require prolonged soaking in the water bath. After the maceration process the bone fragments were removed from the gauze bags and left to dry at room temperature.

To preserve potential microtraces and bone integrity, no degreasing or bleaching procedures were applied afterwards; instead, the specimens were allowed to dry at room temperature. To prevent microbial or fungal growth, the bone fragments were subsequently stored at 8 °C. In previous casework, this procedure has proven to be practical and suitable for preserving potential marks and microtraces.

**Fig. 3:**
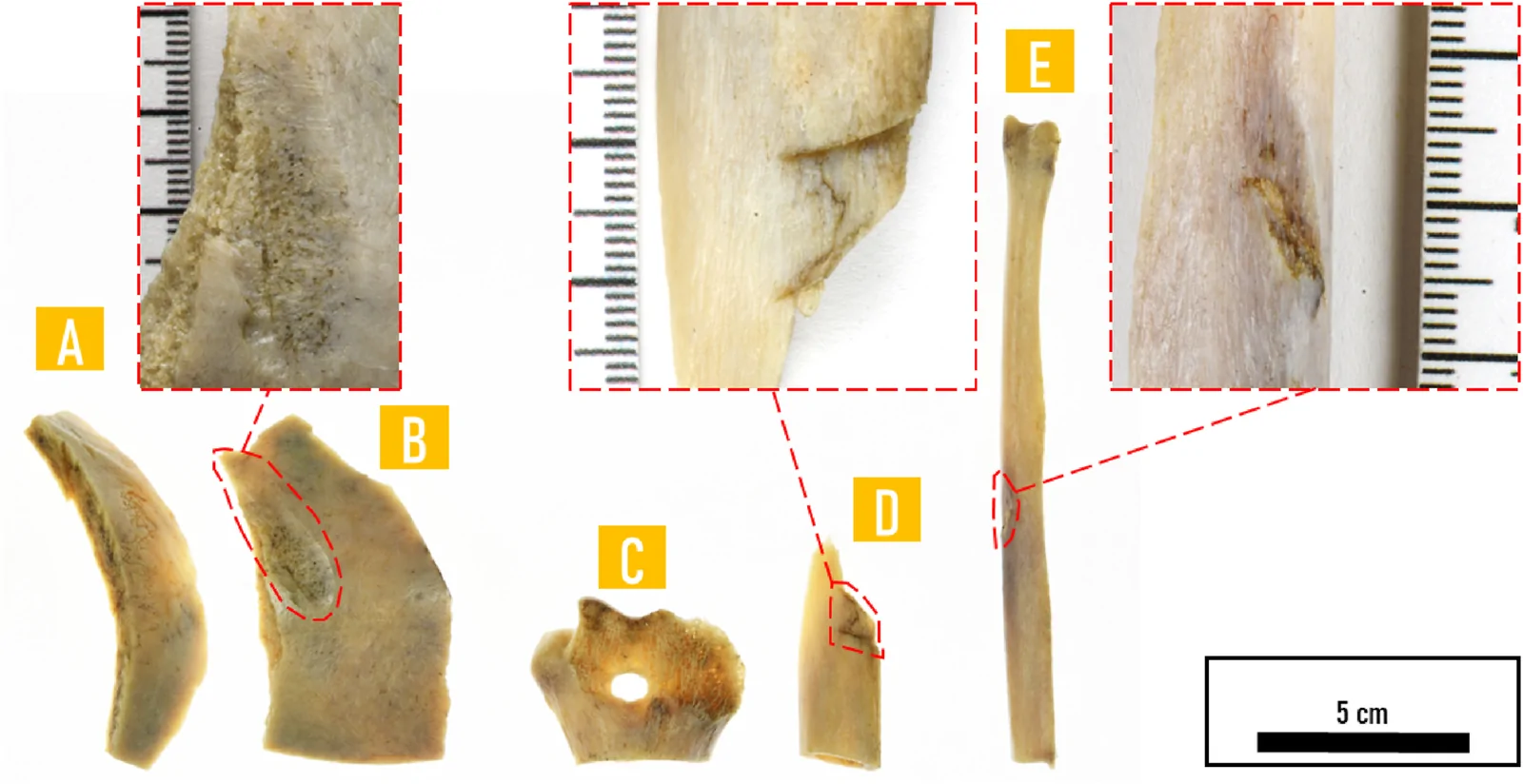
Bone specimens after maceration. A, B skull bone fragments, C right elbow joint fragment, D right humerus fragment, and E left ulna fragment. The tool marks are indicated in B, D and E and shown in enlarged detail.

After maceration, the bones were free of soft tissue and exhibited only minor residual greasiness in isolated areas. Discrete regions bearing toolmarks were identified on the bone fragments from the skull, right humerus and left ulna (Fig. 3).

## 4 Tool marks examination

The public prosecutor requested an examination of the tool marks on the bones of the deceased. The marks were to be compared with a weapon secured at the crime scene. In homicide cases, striation patterns in bone resulting from sharp force trauma can be used to compare both class and individual characteristics with those of a suspected weapon [2], [3], [4], [5].

In general, a mark recovered from a crime scene, in the present case from the bone, is referred to as the questioned mark (QM), whereas a mark experimentally produced with the suspected tool, in the present case a machete, is referred to as a test mark (TM). The basis of a tool marks examination is the assumption that the tool in question possesses features on its working surface that make it unique and distinguishable from other similar tools [2], [4], [3].

Such features arise already during manufacture, for example through grinding, sandblasting, or hardening [6], [7], [8]. These processes produce surface characteristics that are not reproducible with respect to shape, arrangement, number, and size. In addition to these manufacturing-related features, the working surfaces undergo further modifications during use that contribute to their individuality. Scratches, dents, areas of wear, and comparable damage occur depending on the type and intensity of use. When applied to a suitable substrate material, these characteristics are transferred to the resulting mark, for example in the form of striations.

In a toolmark examination, the characteristics of the questioned mark are compared with those of test marks produced using the suspected tool. The resulting observations are evaluated by assessing the evidential value of the findings in light of competing propositions regarding whether the marks originated from the suspected tool or not. This methodological approach is likewise applicable to human tissues such as bone and hyaline cartilage and is frequently employed in forensic casework involving sharp force trauma [9], [10], [11], [12], [13].

**Fig. 4:**
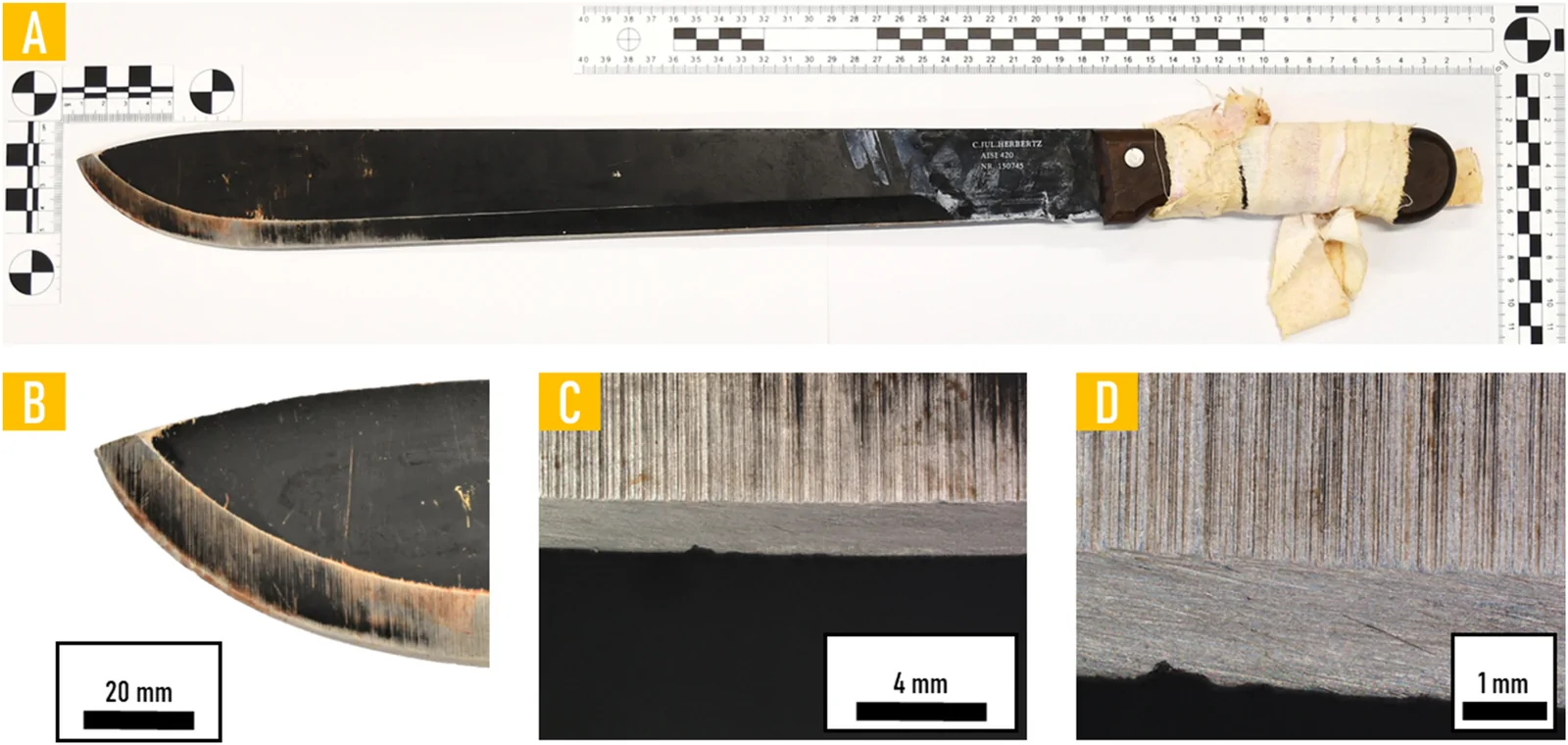
Machete. A: Overview photo (digital photo, Leica D7500), B: Detailed view of the front part of the machete blade (digital photo, Leica D7500). The usage-specific characteristics are clearly visible. C, D Detailed images (digital microscope, Keyence VHX 7000) of the individualizing features (grinding structure and use-related features).

The weapon in question was a machete approximately 600 mm in overall length, with a blade length of approximately 450 mm (Fig. 4). The blade was painted black. Clear signs of use were visible on both the handle and the blade. Furthermore, the cutting edge exhibited sufficient use-related characteristics to render it unique and distinguishable.

**Fig. 5:**
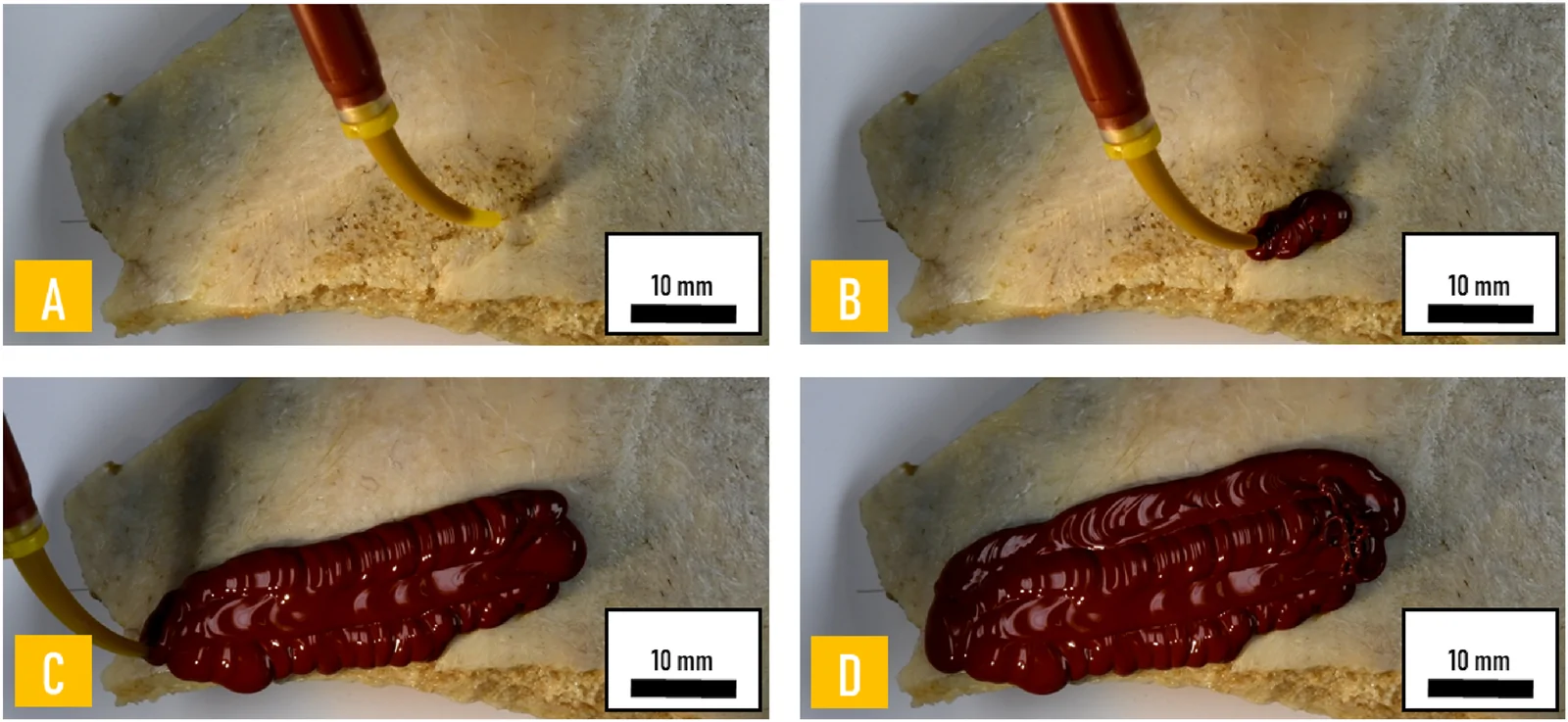
Toolmark recovery by silicone casting of the chop mark on one of the cranial fragments.

Due to the semi-translucent properties of bone, it is often not possible to properly analyze toolmarks by direct examination. To overcome these difficulties, silicone casts of the marks were produced using a brown addition-curing and fast-setting (1 min at 20 °C) silicone material (AccuTrans AB brown, Colténe Wahledent) (Fig. 5) [14], [15], [16].

Test marks were produced using the machete in order compare these with the marks observed in the bone. For this purpose, Modelling Wax plates (Cavex - Set Up Hard) approximately 1 cm in thickness were prepared into which the blade of the machete was carefully struck. Marks were generated along the entire length of the blade and with both sides of the cutting edge. The resulting test marks were subsequently casted with the above-mentioned casting material.

**Fig. 6:**
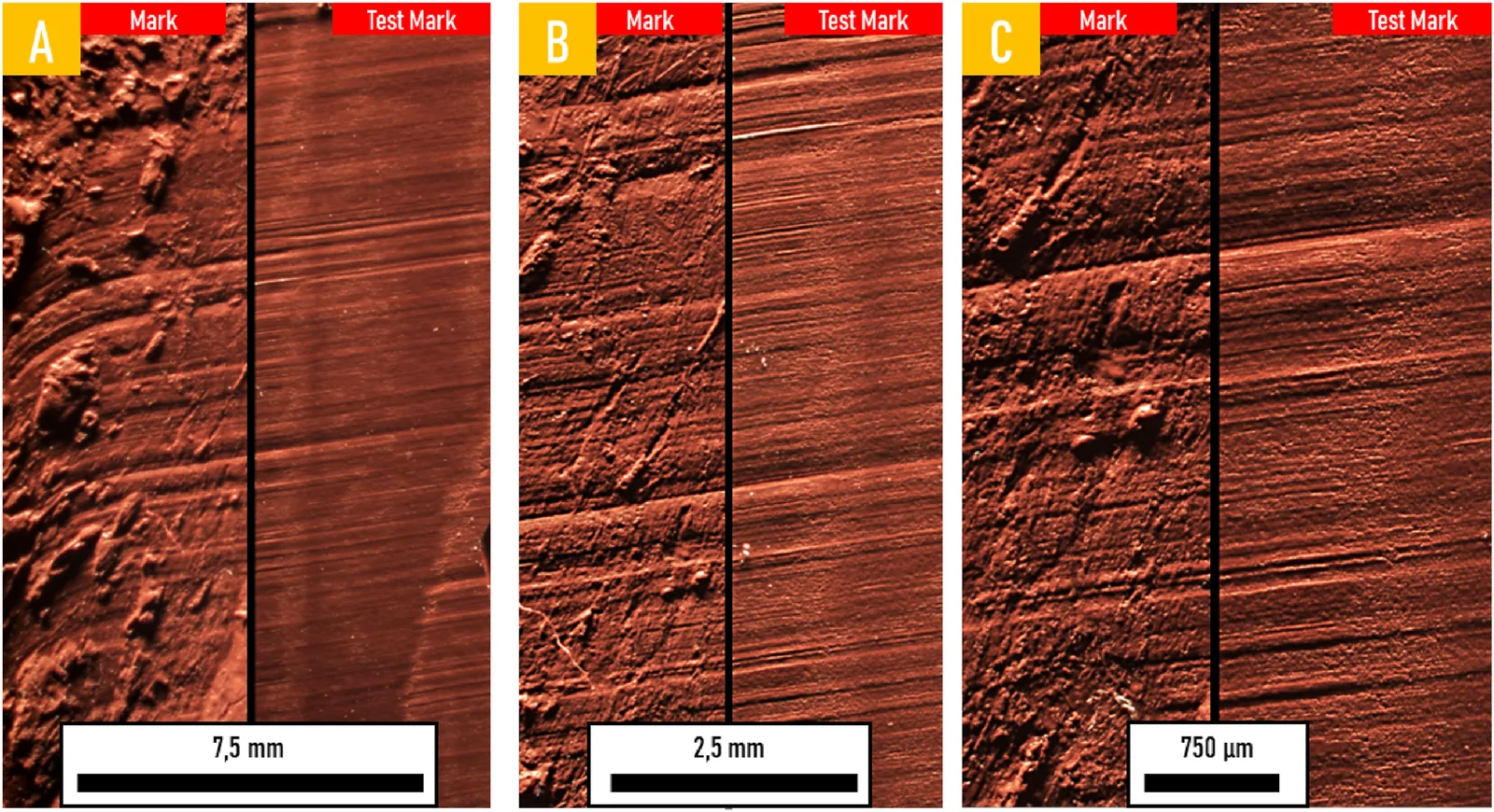
Light microscopic comparison of casts from cranial bone (left) and corresponding test marks (right) at different magnifications (A, B, C).

The casts of the marks from the bone were subsequently compared with those of the test marks. The casts were placed on the stages of a Leica FS-C comparison microscope and analyzed using oblique lighting oriented perpendicular to the striations in order to maximize contrast. Whereas the marks on the right elbow joint, right humerus, and left ulna did not exhibit individualizing features of sufficient quality and quantity to permit comparison, the marks on the skull fragments showed well-defined striations. Comparison with the test marks produced by the machete revealed a high degree of correspondence between the striation patterns of the questioned marks and the test marks (Fig. 6). These observations provide very strong support for the proposition that the questioned and test marks were produced by the same tool (the seized machete) rather than by a different tool.

**Fig. 7:**
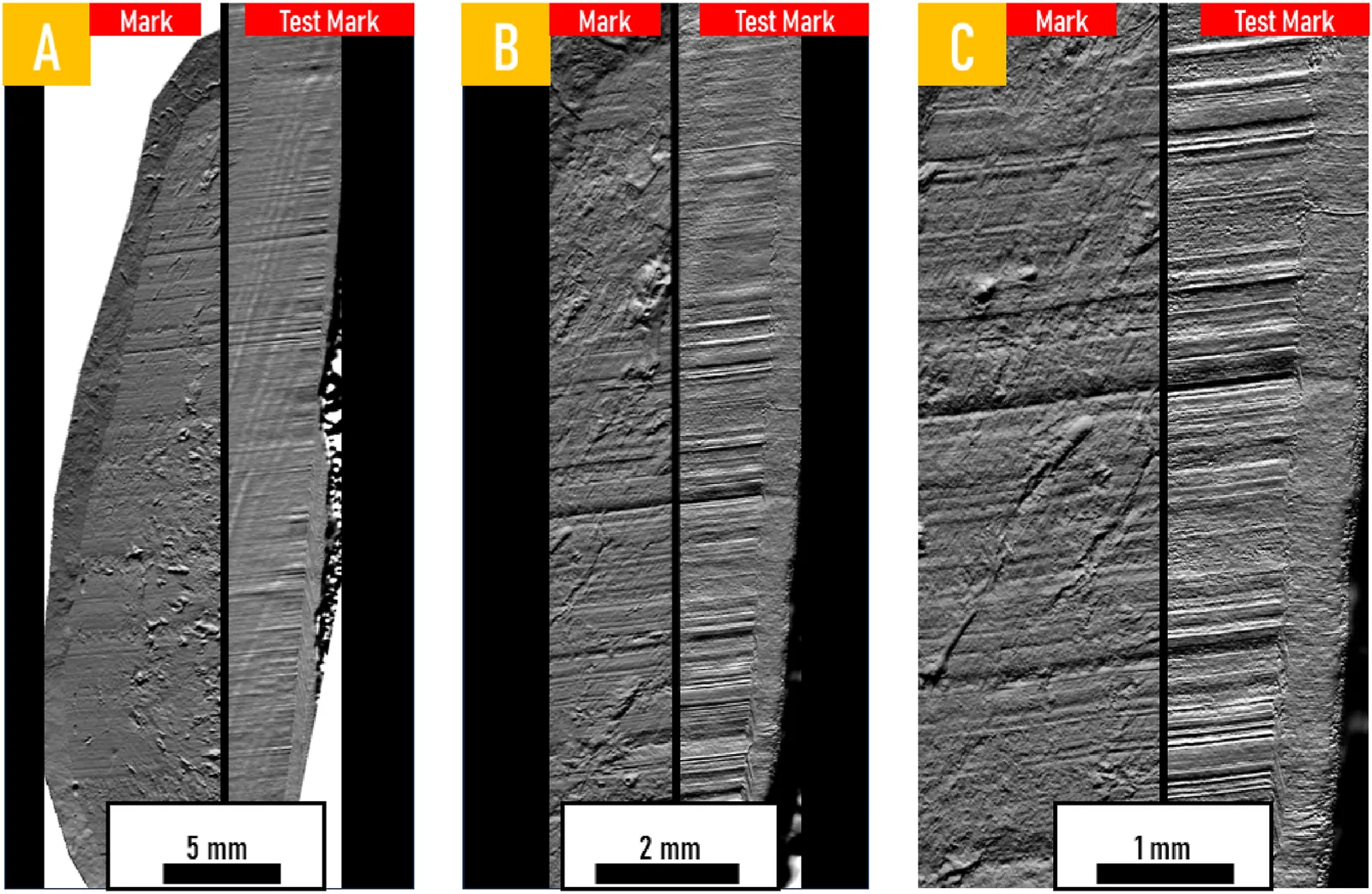
Comparison of the 3D-data of the casts from cranial bone (left) and corresponding test marks (right) at different magnifications (A, B, C).

In addition, casts of the tool marks from the skull bone fragment and the corresponding test marks produced with the machete were digitized using a ToolScan (LIM Laboratory Imaging) forensic 3D scanning system. This system captures macroscopic surface features by laser scanning and combines them with high-resolution microtopography derived from a series of circumferentially illuminated photographs to generate a 3D dataset with a resolution of 3 μm/pixel. The resulting data of the questioned and test marks was subsequently examined using the 3D comparison software LIM Lucia Forensic (ToolScan Version 8.20.1503). The three-dimensional comparison revealed corresponding striation patterns (Fig. 7) consistent with those observed during the light microscopic examination. These findings also provide very strong support for the hypothesis that the questioned and test marks were produced by the same tool.

## 5 Comparison by cross-correlation

Furthermore, the scanned casts were compared numerically using normalized cross-correlation, an approach previously applied to quantify the similarity of striated toolmarks [17], [18]. For this purpose, the existing surface datasets of the questioned mark and the test mark were aligned according to the orientation of the striations, and non-corresponding peripheral areas were removed by cropping. The resulting 3D surface datasets were exported as STL files for subsequent numerical analysis. The STL dataset of the test mark contained 1,921,972 surface points (9.84 mm × 1.83 mm), whereas the questioned mark dataset contained 2,488,086 surface points (9.54 mm × 2.45 mm).

The STL files were imported into a custom-developed MATLAB script (R2024b Update 4, MathWorks, Natick, MA, USA). In the first processing step, each 3D surface dataset was decomposed into a series of individual linear height profiles. A total of 799 profiles were extracted from the mark and 598 profiles from the test mark. During preprocessing, all signals were detrended by subtracting a LOESS^1^-smoothed moving average. This removed low-frequency background components, such as surface waviness and slight tilts of the scanned mark relative to the scanner plane, while preserving the surface features constituting the striation pattern used for comparison. Subsequently, all signals within the mark and test mark were aligned using normalized cross-correlation to compensate for lateral offsets between individual profiles, which may arise from slight curvature of the mark or test mark.

The normalized cross-correlation serves as a measure of the similarity between two signals and is defined by the following equation where k denotes the lag, N the number of discrete data points, and S_1_ and S_2_ the two signatures being compared. S_1_[i] and S_2_[i + k] represent the corresponding height values at index I (Equation (1)).

Equation (1): Normalized Cross Correlation

$$XCorr\left\{k\right\}=\frac{\sum_{i=1}^{N}S_{1}\left[i\right]*S_{2}\left[i+k\right]}{\sqrt{\sum_{i=1}^{N}{\left(S_{1}\left[i\right]\right)}^{2}*}\sum_{i=1}^{N}{\left(S_{2}\left[i+k\right]\right)}^{2}}$$

The denominator normalizes the cross-correlation coefficient, thereby restricting its values to the range between −1 and 1. Consequently, a value of 1 indicates perfect similarity, a value of −1 indicates perfect inverse correlation, and a value of 0 indicates no correlation between the two signatures.

**Fig. 8:**
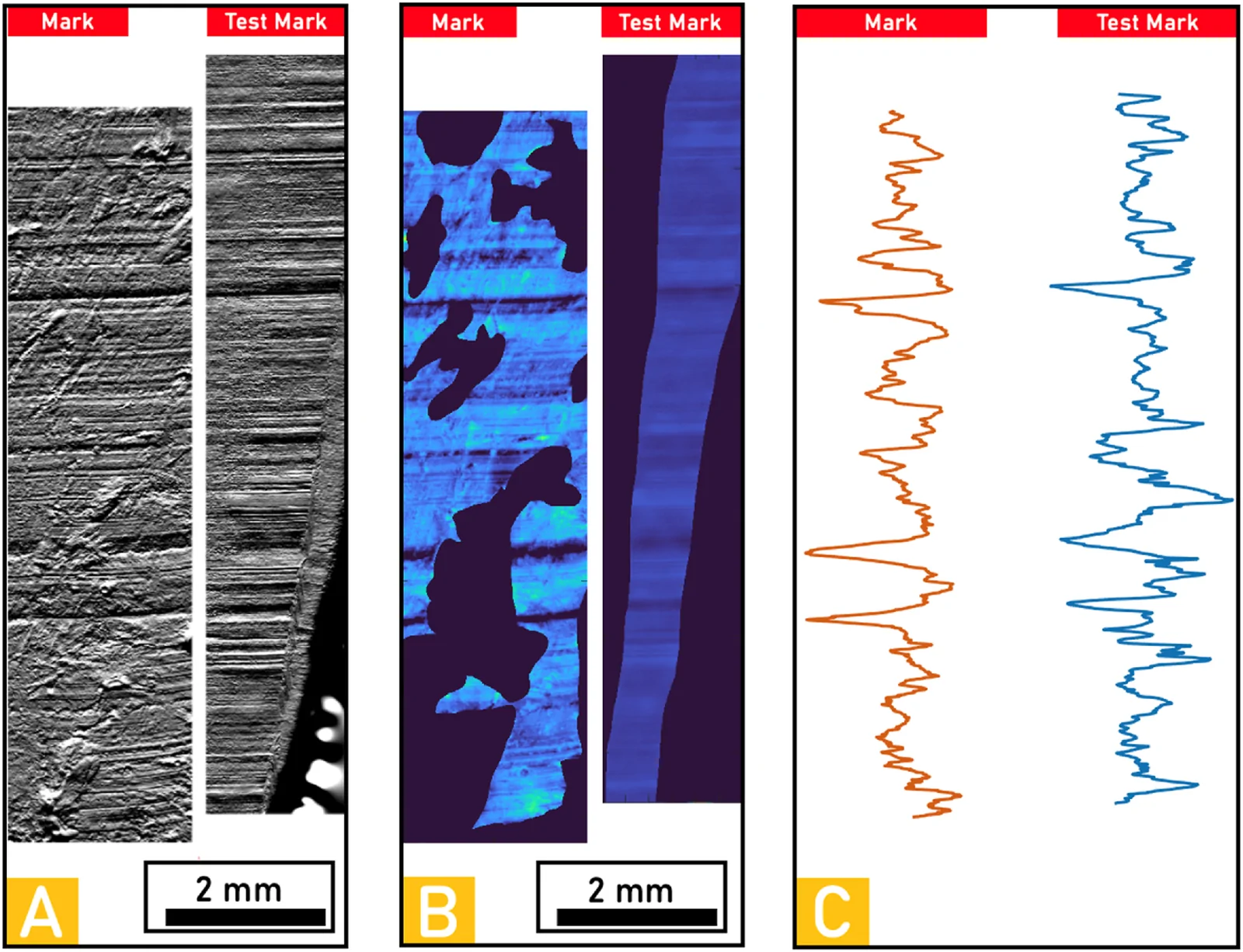
A - Compared areas of the 3D Scan. B - Visualization of the STL surface data in MATLAB after masking non-evaluable regions. C - Resulting representative signatures of mark and test mark.

In a subsequent preprocessing step, non-evaluable regions of the striated marks (e.g. scanning artefacts, contamination, or damaged areas) were manually identified by the examiner, masked, and excluded from further analysis (Fig. 8, A and B). This approach was intended to reflect the principle of conventional optical comparison of striated toolmarks, in which only evaluable striation patterns are considered, while areas without interpretable striations are excluded. Importantly, regions were excluded based on their evaluability rather than on the degree of correspondence between the questioned and test marks.

In contrast to previously described quantitative approaches involving extensive filtering, frequency-band selection, and multi-scale registration [19], [20], preprocessing in the present study was deliberately limited and no frequency-band selection or optimization was performed to maximize the resulting cross-correlation.

**Fig. 9:**
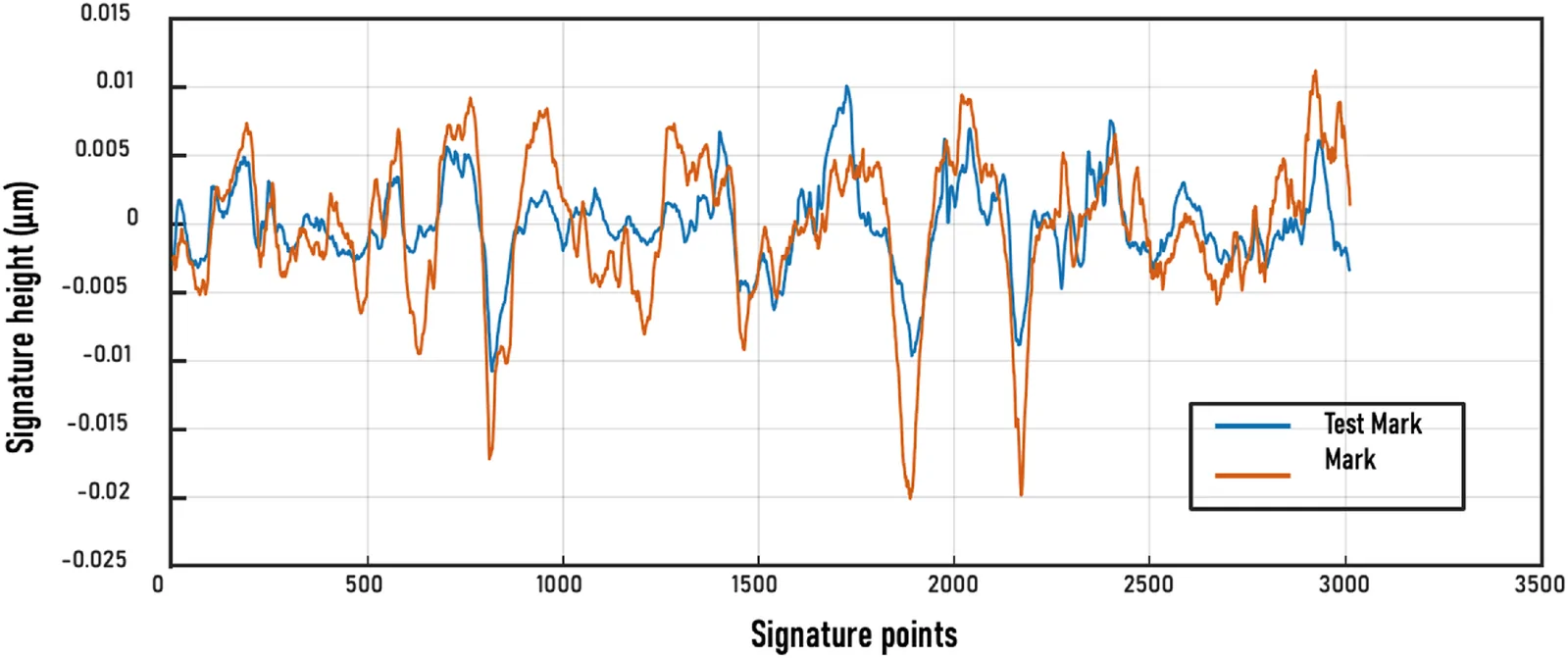
Comparison of the representative signatures of mark and test mark (XCorr 0,73).

Finally, a representative signature was calculated for each comparison and test mark by computing the median across all aligned individual profiles while excluding the previously masked regions (Fig. 8, C). The final step consisted of calculating the normalized cross-correlation between the representative signatures of the comparison mark and the test mark (Fig. 9) which resulted in 0.73.

## 6 Three-dimensional reconstruction and visualization

**Fig. 10:**
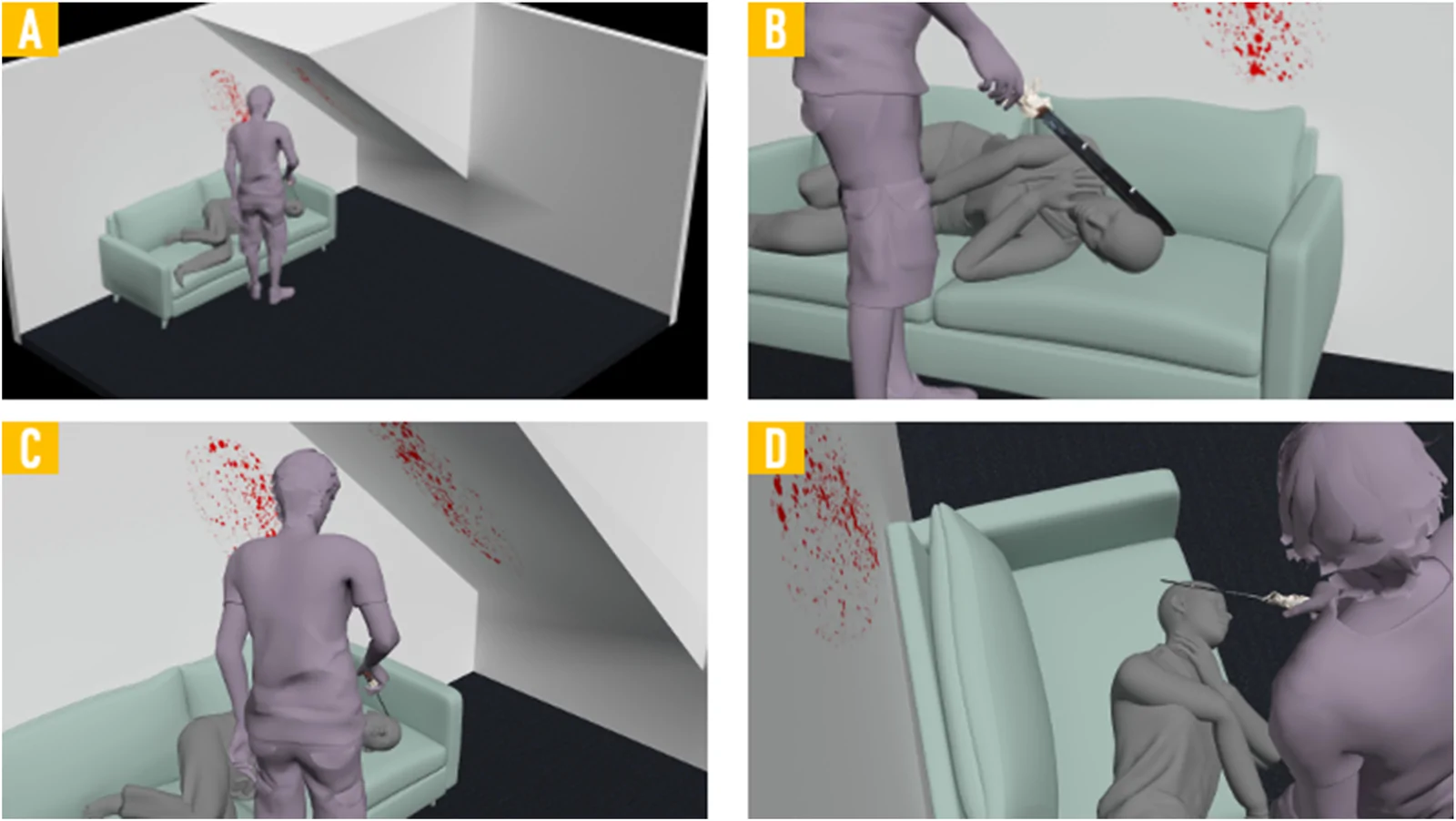
First project; three-dimensional reconstruction illustrating one possible configuration of the crime scene and the relative positions and orientations of the perpetrator and the victim at the time the toolmark was produced. (A) Overview of the crime scene. (B–D) Detailed views from different perspectives. The bloodstains shown on the walls were schematically reproduced based on the findings of the bloodstain pattern analysis and crime scene photographs.

**Fig. 11:**
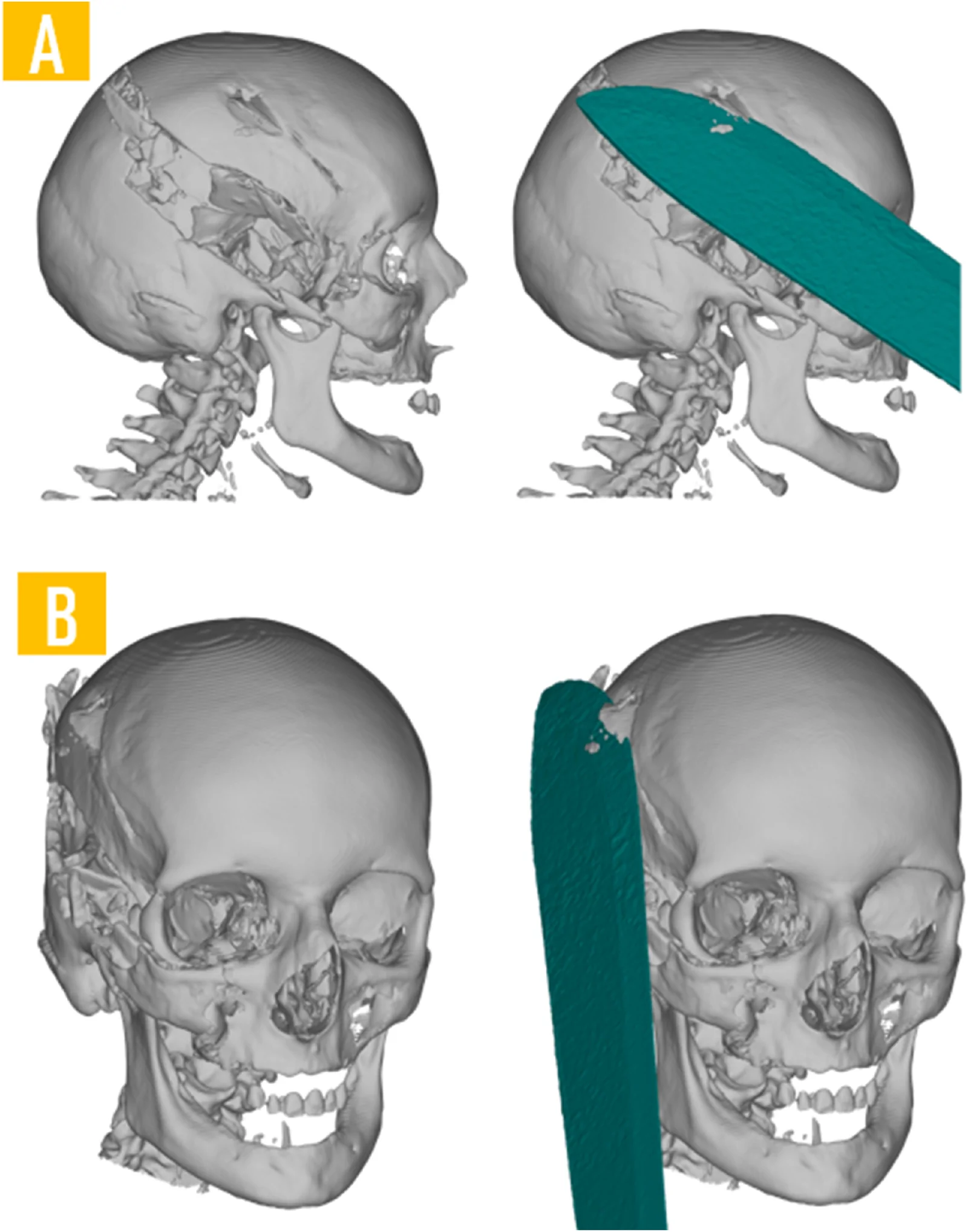
Second project; three-dimensional visualization of the relative orientation of the seized machete and the skull at the time the toolmark was produced. The three-dimensional photogrammetric model of the machete was positioned within the cranial injury according to the results of the toolmark examination. (A) Lateral view and (B) anterior view. In each panel, the pmCT-derived three-dimensional skull model is shown alone on the left and with the aligned machete model positioned within the cranial injury on the right (a three-dimensional model is provided in the supplementary material Fig.B1).

To illustrate one possible configuration in which the tool mark assigned to the machete could have been produced, two complementary three-dimensional projects were created. The first model illustrates the overall crime scene and the relative positions and orientations of the perpetrator and the victim (Fig. 10). The second model provides a detailed visualization of the relative orientation of the machete and the skull at the moment the tool mark originated (Fig. 11).

For this purpose, the data and findings from the case background, forensic medical examination, pmCT data, tool marks examination, bloodstain pattern analysis, photographic crime scene documentation, 3D scans of the crime scene and the three-dimensional photogrammetric model of the crime tool are evaluated and integrated into the model.

The tool marks examination provided several evidence-derived parameters that were directly incorporated into the 3D visualization: the questioned mark was assigned to the left lateral surface of the machete, as the two sides of the cutting edge produced distinguishable striation patterns; the corresponding striation pattern allowed the region of the cutting edge involved in mark formation to be localized with an accuracy of approximately 1 mm; the orientation of the striations defined the axis of blade motion and, in combination with the identified side of the cutting edge and the fact that the striations are produced as the blade enters the bone during the chopping motion, allowed the direction of motion to be determined. Finally, the orientation of the cut surface in the bone determined the orientation of the blade relative to the skull.

For the first project, the crime scene reconstruction, a point cloud acquired with a terrestrial laser scanner served as the basis for the three-dimensional model. Using this dataset, the room geometry and the sofa were reconstructed in Autodesk 3ds Max (version 2024). The seized machete was digitized by means of an object scanner (Botspot BOTSCAN E-COM, Software: RealityCapture 1.4) employing photogrammetric image acquisition. Digital human models representing the individuals involved were obtained from the Mixamo character library and integrated into the virtual scene. In addition, bloodstain patterns considered during the forensic evaluation were schematically incorporated into the scene.

In the second project, the digitally acquired model of the seized machete was visually aligned with the three-dimensional skull model reconstructed from computed pmCT data using Leica Cyclone 3DR. Prior to alignment, the CT dataset was segmented and converted into a standard 3D mesh format using InVesalius (version 3.1), enabling subsequent spatial registration and comparative analysis.

## 7 Discussion

Maceration is a critical step in the examination of toolmarks on bone. Due to the prolonged exposure of the marks to water, elevated temperature, or potentially additives such as enzymes, their surface characteristics may be altered [23], [24]. Therefore, the maceration process should be adapted to the requirements of subsequent examination methods. In case of tool marks, the fine striations must be preserved and should not be altered using harsh chemicals, extreme temperatures or unnecessary exposure to the maceration solution. Cold-water maceration is considered the least harmful method regarding bone integrity and the preservation of the toolmarks. Nevertheless, a major drawback of this method is the enormous amount of time needed to complete the maceration, which may take up to several months. Slightly elevated temperatures (60-65 °C) can accelerate the maceration process by melting the bone fats. But the elevated temperatures do not only come with benefits. Temperatures around 60 °C can denature the proteins present in the bone, potentially resulting in a flaky bone surface and a shrinkage of the bone surface [25] that may hinder toolmark examination. To overcome the drawbacks of both methods, a combination of both maceration methods was applied in this case. Our approach was to combine alternating cycles of warm-water and floating cold-water maceration to gain the benefits of both methods while eliminating their respective drawbacks. Further research is needed to prove that this approach results in a shorter maceration time compared to cold-water maceration and a better preservation of bone integrity and toolmark quality compared to a warm-water maceration alone.

The production of test marks in wax plates proved to be feasible in the present case and enabled the successful comparison with the questioned tool mark. Wax is an established test-mark medium in forensic toolmark examination and has previously been used for the generation and quantitative analysis of striated test marks [3], [5], [19]. Thus, questioned and test marks do not necessarily have to be produced in identical substrate materials, provided that the test medium adequately reproduces the tool characteristics relevant for comparison. However, generating a sufficiently detailed chop mark required numerous repeated strikes before an impression of sufficient quality was obtained. Furthermore, the recording quality of wax is strongly influenced by the material properties, which are highly temperature dependent. At elevated temperatures, the material may deform plastically or smear rather than accurately reproducing fine surface characteristics of the blade as striations. Although bladed tools have previously been successfully identified as the source of chop marks in bone using test marks produced in wax [17], [11], further research is warranted to investigate whether alternative materials may provide more suitable and reproducible substrates for the generation of test marks. In the present study, a new approach was explored in which the principle of conventional optical comparison of striated toolmarks was transferred to the quantitative analysis of three-dimensional surface data. Rather than optimizing the data for maximum correlation, preprocessing was deliberately limited and non-evaluable regions were excluded based on the presence or absence of interpretable striations. The present case should therefore be regarded as an initial assessment of the feasibility of this approach rather than as a validation of its reliability.

**Fig. 12:**
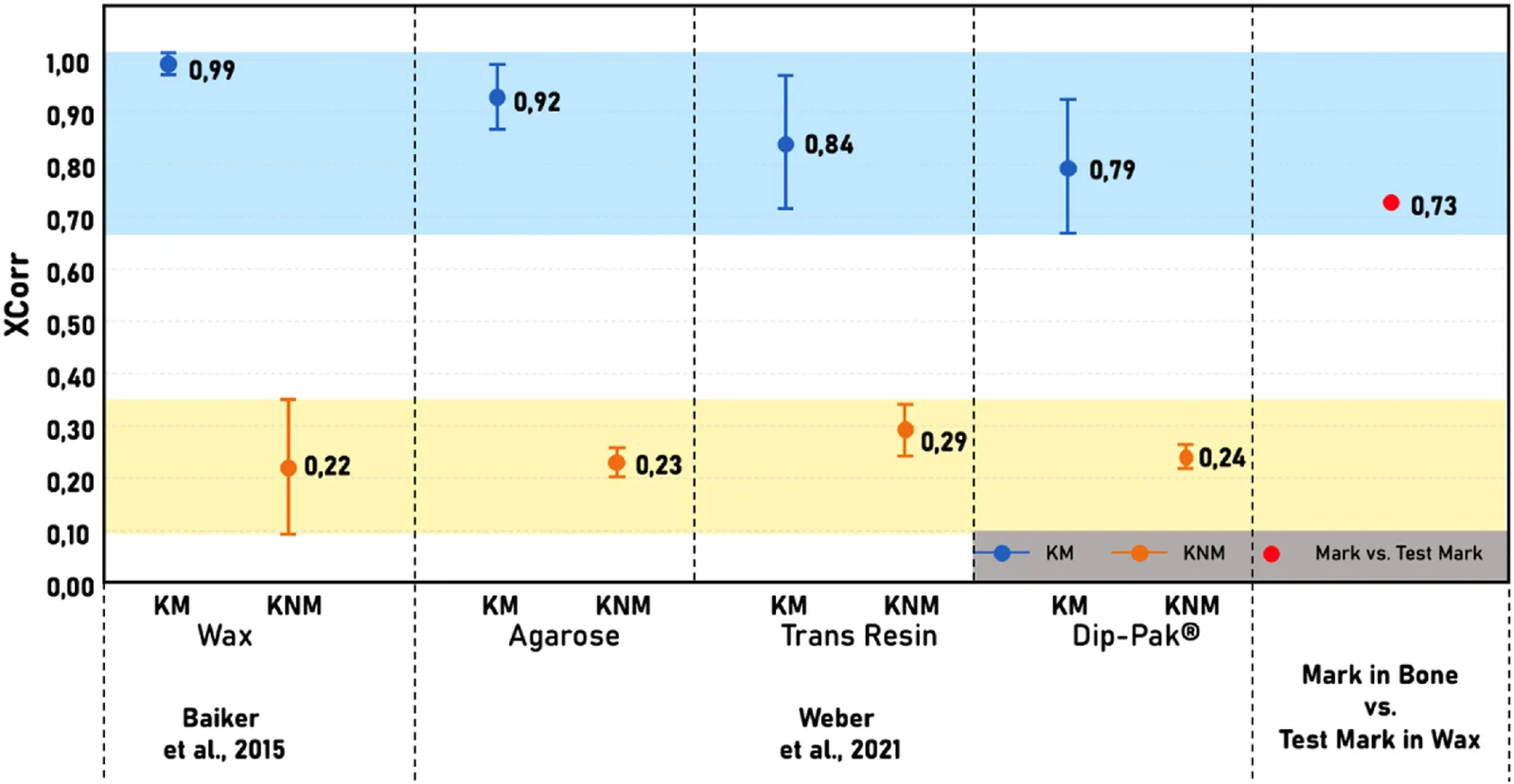
Mean normalized cross-correlation coefficients (XCorr ± SD) for known matches (KM, blue) and known non-matches (KNM, orange) reported by Baiker et al. [21] and Weber et al. [22] for different impression materials under controlled conditions. The red data point indicates the normalized cross-correlation obtained for the comparison of the mark in bone with the corresponding test mark in wax. Shaded areas highlight the ranges of KM (blue) and KNM (yellow) values.

The use of numerical methods for evaluating the similarity between striated marks has been reported in research [19], [20], [21], [26], [27] and casework [17]. However, no universally accepted threshold of normalized cross-correlation exists that reliably distinguishes matching from non-matching tool marks. Baiker et al. [21] compared screw driver marks produced with a motorized tool and calculated a mean cross correlation coefficient for known non matches of 0.22 (STD 0.13) and for known matches of 0.99 (STD 0.01). Weber et al. [22] compared knife cut marks in different materials produced with a custom-made cutting device and calculated cross correlation coefficients depending on the material for known matches between 0.92 (STD 0.006) and 0.79 (STD 0.13) and known non matches between 0.23 (STD 0.03) and 0.29 (STD 0.05). In the present study, a normalized cross-correlation coefficient of 0.73 was obtained (Fig. 12). This value falls within the range of normalized cross-correlation coefficients reported for known matches in the above-mentioned studies, although it is located at the lower end of that range. However, it should be considered that the questioned mark examined here resulted from a homicidal sharp force injury, whereas the marks examined in the referenced studies were obtained under highly controlled and reproducible laboratory conditions using mechanical devices. Given these differences, the relatively correlation coefficient observed in the present study appears plausible and may be regarded as supporting the conclusion that the questioned mark and the test mark were produced by the same tool.

While several parameters of the weapon–skull interaction were directly derived from the toolmark examination, the relative positions and postures of the perpetrator and victim could not be uniquely determined. These aspects of the reconstruction were therefore constrained by the combined forensic findings, including the bloodstain pattern analysis, the location and characteristics of the injuries and other forensic pathological findings, and the documented position and condition of the deceased at the scene. Consequently, the resulting 3D visualization represents one possible configuration consistent with the available evidence rather than a unique reconstruction of the event.

The quality of any forensic reconstruction fundamentally depends on the completeness and accuracy of the documented crime scene, trace evidence, and injuries sustained by the deceased or survivors [28]. For comprehensive three-dimensional reconstruction and visualization, it is essential that the crime scene is documented using high-quality 3D acquisition methods with sufficient spatial resolution. A complete and accurate dataset provides greater flexibility during subsequent reconstruction and visualization, allowing different perspectives, hypotheses, and analytical approaches to be explored without the need for additional scene acquisition. In contrast, physical evidence that has been secured and preserved, such as weapons or other exhibits, can be digitized retrospectively without compromising the reconstruction process. Therefore, immediate 3D acquisition of such items is less critical.

A digital model of a suspected weapon offers additional practical benefits, as physical replicas can be produced by 3D printing and used for demonstration in court or for direct comparison with injuries during autopsy [29]. As a contact-free alternative, virtual fitting of the weapon to the injury may be performed in a three-dimensional or virtual reality environment. This approach avoids potential contamination with trace material and eliminates the risk of altering toolmarks through physical contact with a 3D-printed replica.

Beyond the identification of the weapon, this study demonstrates the potential of integrating different forensic disciplines into a single reconstruction. Crime scene documentation, forensic autopsy, pm CT imaging, bloodstain pattern analysis and tool mark examination each contributed complementary information that, when combined, enabled a detailed reconstruction of the weapon position and orientation at the moment the tool mark assigned to the machete was produced. The resulting three-dimensional models provide a natural visualization of the complex forensic findings.

Such interdisciplinary reconstructions have considerable potential for future forensic casework. They may support criminal investigations by enabling the comparison of reconstructed events with witness statements and other investigative findings. Furthermore, they may facilitate the presentation of complex forensic evidence in a clear and comprehensible manner during criminal proceedings.

Establishing standardized workflows for the early exchange of relevant data—including pm CT datasets, three-dimensional crime scene documentation, bloodstain pattern analysis and tool mark examination results— may facilitate the integration of findings from different forensic disciplines in future reconstructions.

The present case demonstrates the feasibility of integrating findings from multiple forensic disciplines into a comprehensive three-dimensional reconstruction. Future investigations of comparable cases may benefit from early coordination between the involved disciplines to ensure the timely acquisition and exchange of all relevant data required for a comprehensive reconstruction.

## CRediT authorship contribution statement

**Melissa Kirbach:** Writing – original draft, Visualization, Software, Methodology, Data curation, Conceptualization. **Anna Holzer:** Writing – original draft, Investigation. **Larissa Frey:** Writing – original draft, Visualization. **Anna Tegou:** Writing – original draft, Visualization. **Sven Helmrich:** Visualization. **Markus Alexander Rothschild:** Writing – review & editing. **Matthias Weber:** Writing – review & editing, Writing – original draft, Visualization, Supervision, Software, Methodology, Investigation, Data curation, Conceptualization.

## Declaration of generative AI use

During the preparation of this work, the authors used OpenAI ChatGPT (GPT-5.5) to improve the language, grammar, style, and clarity of the manuscript and to assist with editorial revisions. After using this tool, the authors reviewed and edited all generated content as needed and take full responsibility for the content of the published article.

## Funding

Open Access funding enabled and organized by Projekt DEAL.

## Declaration of competing interest

The authors declare that they have no known competing financial interests or personal relationships that could have appeared to influence the work reported in this paper.

## Footnotes

1 LOESS (Locally Estimated Scatterplot Smoothing) is a non-parametric local regression method for estimating smooth trends in noisy data.
