# Supplementary material for: Signed in bone: Toolmark identification of a machete and multidisciplinary 3D reconstruction in a homicide investigation
Source: Forensic Sci Int Synerg. 2026 Sep 18;13:100739. doi: 10.1016/j.fsisyn.2026.100739 (PMC13613511; doi:10.1016/j.fsisyn.2026.100739)
Supplement: Supplementary file 1 [file mmc1.pdf]

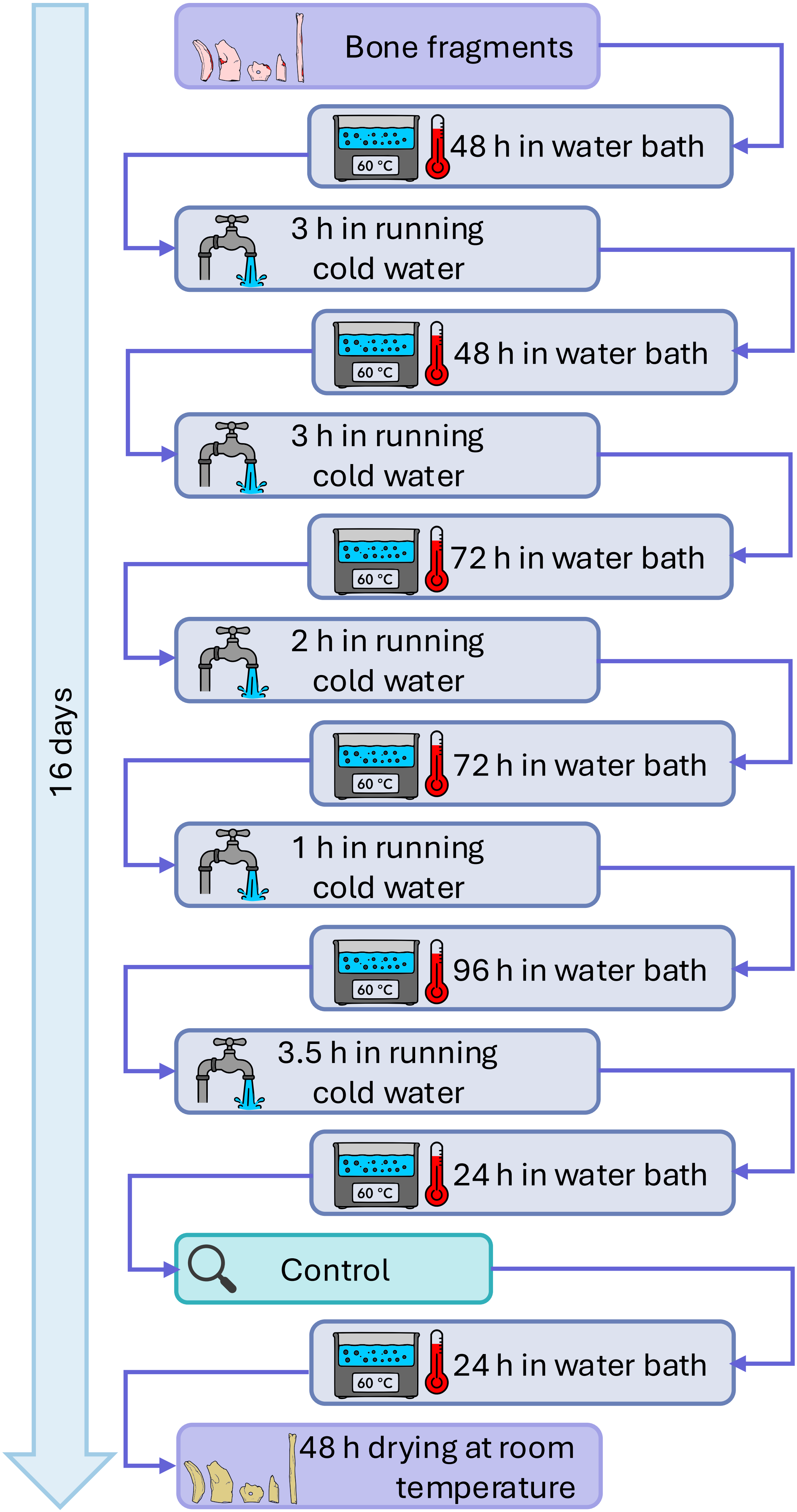

16 days
Bone fragments
60 °C
48 h in water bath
3 h in running cold water
60 °C
48 h in water bath
3 h in running cold water
60 °C
72 h in water bath
2 h in running cold water
60 °C
72 h in water bath
1 h in running cold water
60 °C
96 h in water bath
3.5 h in running cold water
60 °C
24 h in water bath
Control
60 °C
24 h in water bath
48 h drying at room temperature
